# Intestinal Amyloidosis in Common Variable Immunodeficiency and Rheumatoid Arthritis

**DOI:** 10.1155/2015/405695

**Published:** 2015-08-16

**Authors:** T. Meira, R. Sousa, A. Cordeiro, R. Ilgenfritz, P. Borralho

**Affiliations:** ^1^Gastroenterology Department, Hospital Garcia de Orta E.P.E., Avenida Torrado da Silva, 2801-951 Almada, Portugal; ^2^Rheumatology Department, Hospital Garcia de Orta E.P.E., Avenida Torrado da Silva, 2801-951 Almada, Portugal; ^3^Anatomical Pathology Department, Hospital Garcia de Orta E.P.E., Avenida Torrado da Silva, 2801-951 Almada, Portugal

## Abstract

We present a case of reactive amyloidosis that developed secondary to common variable immunodeficiency and rheumatoid arthritis. A 66-year-old woman, with prior history of common variable immunodeficiency and rheumatoid arthritis, was referred to our clinic for chronic diarrhea investigation. The patient was submitted to colonoscopy with ileoscopy, which did not show relevant endoscopic alterations. However, undertaken biopsies revealed amyloid deposition. Since amyloidosis with GI involvement is a rare cause of chronic diarrhea, this pathology should be considered in etiologic investigation, especially when associated with chronic inflammatory diseases.

## 1. Introduction

Amyloidosis is a rare disease resulting from extracellular deposition of insoluble fibrillar proteins, subclassified as primary when fibrils result from monoclonal light chain fragments deposition and as secondary when the accumulated material is serum amyloid A [1]. In the past, the main cause for AA amyloidosis was chronic infectious diseases, such as tuberculosis. Nowadays, 50% of cases are due to chronic inflammatory diseases, with rheumatoid arthritis (RA) being the most frequent one, followed by ankylosing spondylitis and psoriatic arthritis [2].

In secondary subclassification, amyloidosis histological gastrointestinal (GI) involvement is very common, although clinically overt disease is rare [2]. On the contrary, in primary amyloidosis GI involvement occurs less frequently, once only 8% have amyloid tissue infiltration and 1% of patients have GI symptoms [3].

We report an unusual case of intestinal AA amyloidosis in a patient with common variable immunodeficiency and rheumatoid arthritis.

## 2. Case Report

**Figure 1:**
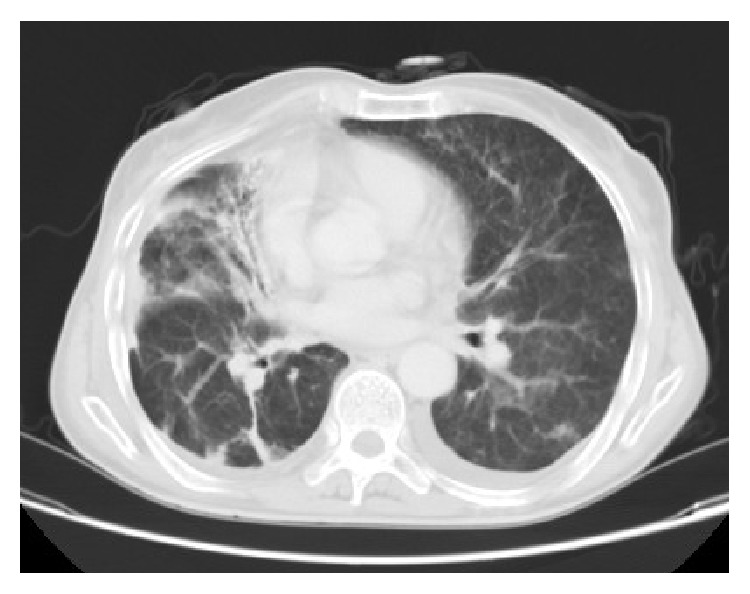
Chest computed tomography: bilateral interstitial thickening and bronchiectasis in the right hemithorax.

A 66-year-old female with history of pleuropulmonary tuberculosis in 1979 and a thymoma in 2006 was submitted to resection and adjuvant radiotherapy. Since 2006, the patient has been investigated for recurrent respiratory tract infection. According to the complementary investigation conducted, our patient was submitted to a chest computed tomography, which showed bilateral interstitial thickening and bronchiectasis in the right hemithorax (Figure 1), leading to the conclusion of being radiation pneumonitis.

Also in 2006, she was referred to Rheumatology clinics for arthritis and rheumatoid factor and antinuclear antibody positives. Laboratory studies revealed the following: high serum value of C-reactive protein (CRP) 22 mg/dL (normal range: <0.1) and decreased immunoglobulins, IgG 593 mg/dL (normal range: 700–1000), IgA 40 mg/dL (normal range: 70–400), and IgM 8 mg/dL (normal range: 40–230). After exclusion of other rheumatic conditions, the diagnosis of rheumatoid arthritis was assumed. Due to high articular activity, immunomodulation was introduced starting with increasing doses of sulphasalazine, up to 3 g per day (patient refused methotrexate), hydroxychloroquine, naproxen up to 1000 mg per day, deflazacort 6 mg/day, alendronic acid 70 mg/week, and calcium, resulting in partial improvement of arthritis. She was referred to Hematology that confirmed the diagnosis as Common Variable Immunodeficiency, initiating a monthly treatment with intravenous human immunoglobulin.

**Figure 2:**
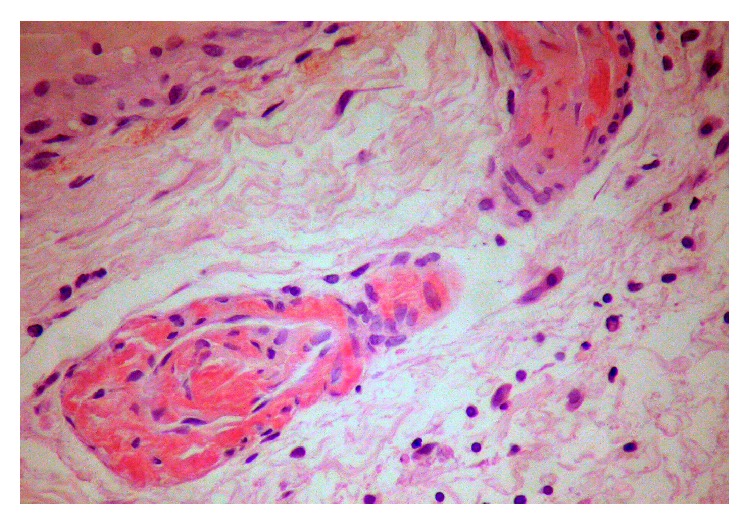
Perivascular amyloid deposit in colon submucosa (Congo red staining without polarization).

**Figure 3:**
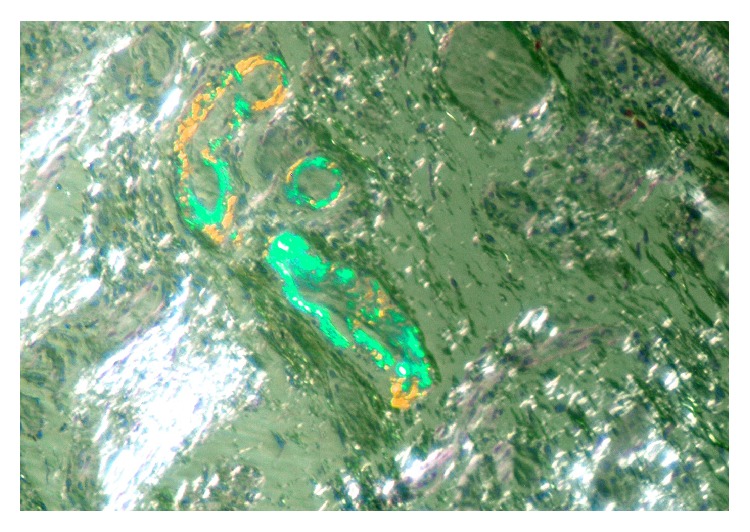
Perivascular amyloid deposit in colon submucosa (Congo red staining, with polarization).

**Figure 4:**
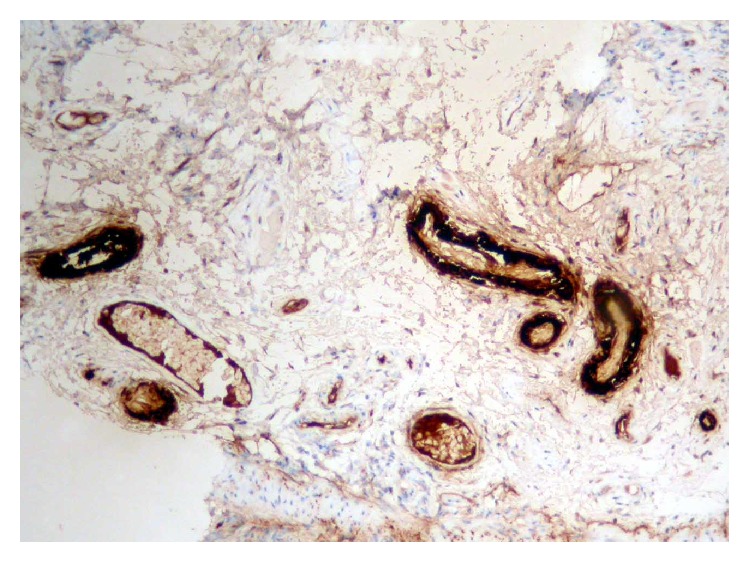
Immunolabeling pattern with monoclonal anti-amyloid A antibody (mc1 clone).

Two years after the diagnosis, the patient had worsened articular activity and developed watery diarrhea, with average stools frequency of up to 4 to 5 times a day with no blood, mucus, or pus, along with intermittent abdominal pain. She denied fever, hematic losses, anorexia, weight loss, and profuse sweating. Abdominal physical examination was unremarkable and laboratory findings revealed the following: no anemia, thrombocytosis of 487000 platelets/L (normal values: 120–44000), erythrocyte sedimentation rate (ESR) 28 mm/h (normal value: 0–15), and 3.6 mg/dL of CRP. Microbiologic and parasitological analyses of the stools were negative. HIV and anti-CMV IgM serologies were negative. The colonoscopy with ileoscopy showed an accentuated vascular pattern along the colon, with no other significant changes. Biopsies were obtained from different colon segments. Microscopically, deposition of amorphous hyaline material that infiltrated the submucosa wall in colon with hematoxylin-eosin was observed. Congo red staining allowed detection of green birefringence on fibrils (Figure 2). Immunohistochemistry showed marking of AA proteins, confirming a reactive amyloidosis (Figures 3 and 4). Additional investigation was performed having excluded renal involvement by amyloidosis, with no changes in proteinuria and creatinine clearance.

The patient became asymptomatic after controlling RA activity with the addition of Tocilizumab, a biologic agent (TB reactivation excluded: chest CT, bronchofiberscopy, and cultures), and has been kept under surveillance without GI symptoms.

## 3. Discussion

Common variable immunodeficiency (CVID) is the most symptomatic primary antibody deficiency, characterized by hypogammaglobulinemia, leading primarily to recurrent pulmonary or GI infections [4]. Such as in our case, where the patient had recurrent pulmonary infection after treatment for thymoma, these complications may be present at the onset or may appear later.

In about 20% of the cases, common variable immunodeficiency is also associated with autoimmune diseases, such as rheumatoid arthritis [5]. In our case, the RA diagnostic was established simultaneously with CVID. While the pathogenesis of autoreactivity is unknown for CVID subjects in general, and to a greater extent for those with autoimmunity, there is a loss of switched memory B cells [5].

The incidence of gastrointestinal diseases in CVID group has a considerable relevance, referred to be between 20 and 60%, with the chronic diarrhea being the most common presentation of this disease [6]. Between 6 and 10% of CVID patients develop inflammatory bowel disease-like disorder and a poor group develop celiac enteropathy like lymphoma, gastric adenocarcinoma, and intestinal amyloidosis. The alterations on immune mediators such as impaired antibody production, disruption on T-cell function, and defects in innate immunity could be responsible for GI disease observed in CVID patients [6]. The secondary amyloidosis is a rather rare complication of the CVID; a time frame of 8 to 14 years between the AA amyloidosis diagnostic and the CVID inflammatory condition is seldom observed [7]. All amyloidosis CVID cases have recurrent infections, which was also observed in our patient, consisting in a relevant condition supposed to be related to amyloidosis progression [7]. The intravenous immunoglobulin administration decreased our patient's episodes of pulmonary infections but not the GI symptoms.

Recently, LRP12 mutations were found in patients with intestinal amyloidosis and CVID, which denotes the complex association between immunodeficiency and autoinflammatory disease [8].

Rheumatoid arthritis can either be associated with CVID or be an independent condition for amyloidosis development, which was not possible to determine in our case. Nowadays, RA is known to be one of the most common causes of AA amyloidosis, with a prevalence between 10 and 29% of patients with such disorder, varying according to different previous studies [9], usually associated with long duration (7 to 10 years) and uncontrolled rheumatoid arthritis. Our patient had a short duration and nonerosive arthritis at amyloidosis diagnosis, which however could have been a subclinical disease. Rheumatoid arthritis therapeutics aims to achieve disease remission by suppressing inflammation and immunological dysregulation [10]. Sulphasalazine was initially chosen instead of methotrexate, either due to the patient's refusal of that drug or due to the potentially limiting pulmonary drug toxicity. However, articular inflammatory activity was recurrently observed, requiring a more potent therapeutic approach.

Due to the history of pulmonary tuberculosis and immunomodulator therapy, mycobacterium intestinal infection should be excluded. The patient did not present clinical evidence of pulmonary tuberculosis and had normal chest X-ray. However, 76% of intestinal tuberculosis cases may occur in asymptomatic patients. The absence of granuloma or alcohol-acid-resistant bacilli at colonic biopsy leads to disregarding such hypothesis [11].

Independently of the trigger for secondary AA, there are unspecific gastrointestinal symptoms, varying from gastrointestinal haemorrhage to chronic intestinal dysmotility, or malabsorption caused by colon mucosa infiltration and bacterial overgrowth, or even exudative gastroenteropathy. Its endoscopic appearance derives from colon mucosa infiltration, more frequent at the descendent duodenum followed by stomach, colon, and esophagus. Intestinal mucosa may present itself slightly granular and friable erosions, ulcerations, and submucosa hematomas; nevertheless, polypoid or nodular lesions can rarely occur [1, 12]. Intestinal biopsies are diagnostic of AA amyloidosis in 75–95% of cases and it should be performed even in the presence of normal appearing mucosa [13].

Several studies have focused on genetic factors, with prognostic significance, which might increase susceptibility to AA, aiming to identify patients that would benefit from more aggressive treatments. To date, SAA genotype is the only established variable that significantly affects the risk of AA development. SSA genotype was not evaluated in our patient [14].

The aim of symptomatic treatment is to control the underlying disease. In cases of severe diarrhea and exudative gastroenteropathy, the use of steroids, octreotide, or immunosuppressors should be considered [14].

Our patient had two pathologic entities that can be associated with amyloidosis, common variable immunodeficiency and rheumatoid arthritis, both contributing to the development of intestinal amyloidosis. The perception of which pathology had a more significant contribution to amyloid substances intestinal deposition was unachievable. However, the previous history of pulmonary tuberculosis, along with recurrent pulmonary infections during the course of CVID and the CVID itself which led to RA development, may be in the origin of the amyloid deposition.

In conclusion, when investigating unknown etiology of chronic diarrhea, associated with chronic inflammatory disease, the biopsies are essentials, permitting the diagnosis of rare intestinal amyloidosis, allowing a clinical and therapeutic estimate, and significantly contributing to patient's quality of life.
